# Visual prognosis in idiopathic intracranial hypertension: observations from a retrospective cohort in Germany

**DOI:** 10.3389/fneur.2025.1698486

**Published:** 2025-11-12

**Authors:** Theresia Knoche, Lisa Varlet, Anne Pohrt, Leon Alexander Danyel, Paula Haffner, Alexander Bernhard Kowski

**Affiliations:** 1 Department of Neurology, Charité – Universitätsmedizin Berlin, Berlin, Germany; 2 Berlin Institute of Health at Charité – Universitätsmedizin Berlin, Berlin, Germany; 3 Institute for Biometry and Clinical Epidemiology, Charité – Universitätsmedizin Berlin, Berlin, Germany

**Keywords:** idiopathic intracranial hypertension, outcome, prognosis, visual outcome, papilledema, Pseudotumor Cerebri

## Abstract

**Background:**

Idiopathic intracranial hypertension (IIH) is a rare disorder of raised intracranial pressure that can cause visual loss. While risk factors for visual outcome have been explored in international cohorts, no data are available from Germany. This study is the first to evaluate clinical characteristics and predictors of visual deterioration in a large German cohort of IIH patients.

**Methods:**

We retrospectively analyzed patients diagnosed with IIH between 2004 and 2020 at a tertiary neurological center. Clinical features, ophthalmologic findings, and treatment strategies were recorded. Visual outcomes were assessed at minimum follow-up of 6 months after IIH diagnosis. Poor visual outcome was defined by worsening of visual function or persistent visual impairment. Regression analysis was utilized to evaluate potential risk-factors of poor visual outcome.

**Results:**

The cohort included 191 consecutive IIH patients; follow-up ophthalmologic data were available in 90. Poor visual outcome occurred in 30%. Multivariable regression showed male sex (OR 8.7, *p* = 0.009) and severe papilledema at baseline (OR 7.7, *p* = 0.02) were independently associated with poor outcome. Age, disease duration, BMI, and CSF opening pressure were not predictive.

**Conclusion:**

Our findings confirm papilledema severity and male sex as prognostic factors identified in prior studies and extend them to a German real-world setting. This strengthens the external validity of existing evidence and underscores the importance of early recognition of high-risk patients to prevent irreversible visual loss.

## 1 Introduction

Idiopathic intracranial hypertension (IIH) is characterized by visual disturbance and burdening headaches due to increased intracranial pressure (ICP). It predominantly affects young overweight and obese women, with weight gain dynamics recognized as an important risk factor (1). The exact etiology remains unknown. The primary therapeutic goal in IIH is the prevention of permanent visual loss, alongside relief of symptoms. However, predicting the visual prognosis remains challenging due to the rarity of the condition and its heterogenic clinical course. While some patients achieve remission under dietary measures and ICP-lowering medication, others with more severe disease may require neurosurgical or endovascular interventions. Identifying prognostic factors at the time of diagnosis is therefore crucial to guide individualized treatment strategies.

Previous studies have linked poor visual outcomes to male sex, longer disease duration, severe papilledema, and reduced visual acuity at baseline (2–4). Cerebrospinal fluid (CSF) opening pressure has been reported as a potential predictor (5), although findings have been inconsistent (2, 6). Magnetic resonance imaging (MRI) features, in contrast, do not appear to reliably predict headache or visual outcomes (6). Recently, neurofilament light chain has emerged as a biomarker of axonal damage and neurodegeneration, showing associations with papilledema severity and visual field defects in IIH (7, 8). The longitudinal course of IIH has so far been described mainly in a few large prospective cohorts, often focusing on specific interventions or using heterogeneous diagnostic criteria. The diagnostic framework was recently refined by Friedman et al., whose criteria have since been incorporated into current guidelines (9, 10). To the best of our knowledge, baseline characteristics, visual outcomes, and risk factors of visual loss have not yet been systematically assessed in a German IIH cohort.

The aim of this study was to characterize the clinical profile and visual outcomes of IIH in the first German cohort and to identify predictors of visual loss in this population.

## 2 Materials and methods

This is a single-center cohort study evaluating factors that predict poor visual outcome in a retrospectively collected cohort of IIH patients. All patients were treated at the neurological department of Charité – Universitätsmedizin Berlin, which is a tertiary care university hospital. This research study was conducted retrospectively from data obtained for clinical purposes. The need for informed consent was waived and retrospective data extraction and analysis was performed in approval with the ethics committee of Charité – Universitätsmedizin Berlin, Germany, on 12 February 2021 (Application No. EA4/004/21). This study complies with the reporting guidelines within the Strengthening the Reporting of Observational Studies in Epidemiology (STROBE) Statement.

### 2.1 Patient selection

**Figure 1:**
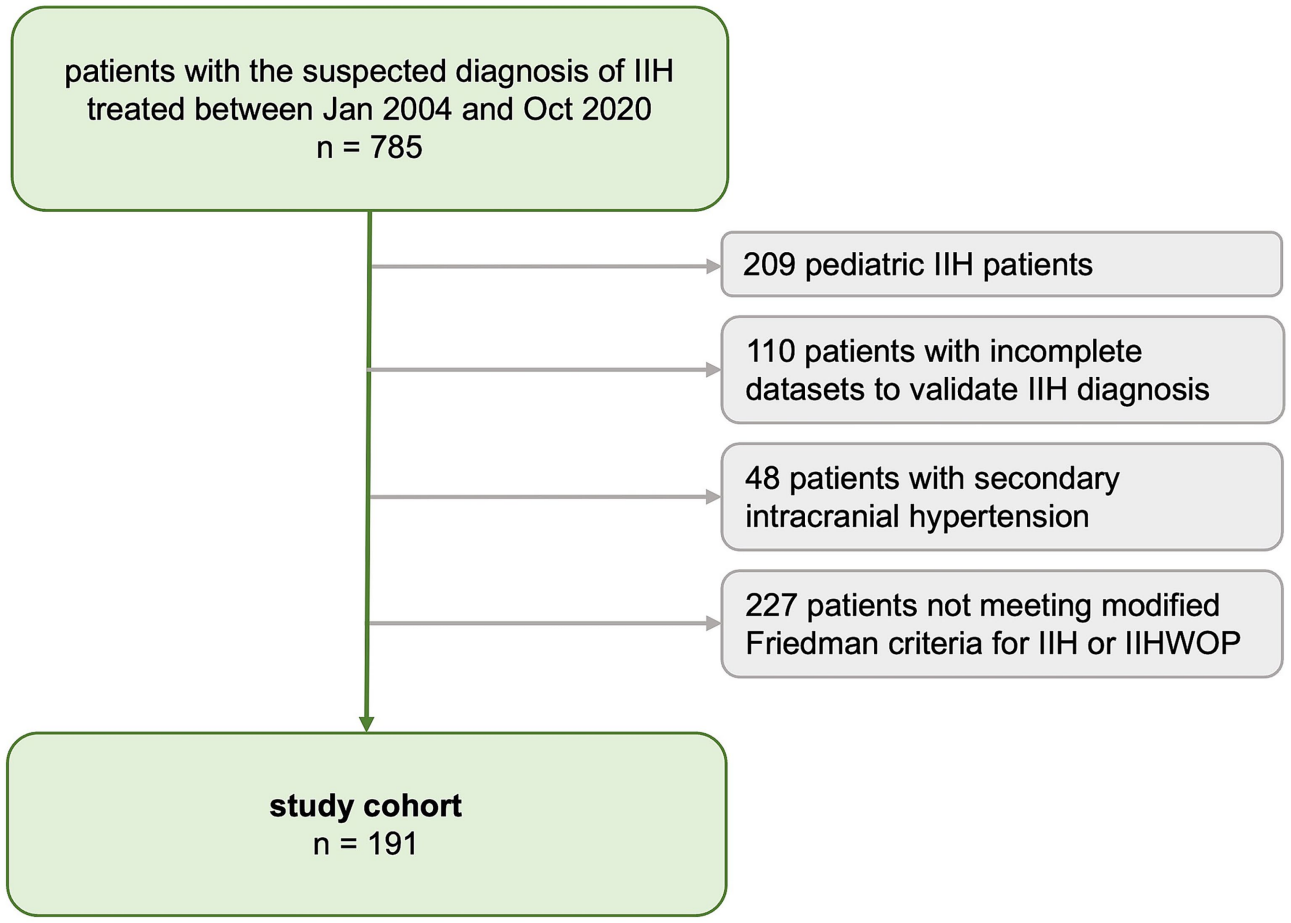
Flow chart of the inclusion/exclusion process.

A medical database inquiry identified all consecutive patients treated under the definite or suspected diagnosis of IIH (ICD-10 code: G93.2) at our tertiary care center between January 2004 and October 2020. Individual patient charts were reviewed to determine whether the diagnosis of IIH could be verified based on the revised Friedman criteria for IIH (10). For the present study, we included all patients with definite IIH and IIH-WOP (IIH without papilledema) according to Friedman criteria. Patients were excluded if data to determine the diagnosis of IIH was insufficient (i.e., lack of ophthalmologic examination conducted prior to lumbar puncture, or lack of MRI for exclusion of secondary causes or measurement of CSF opening pressure (CSF-OP)) or if the IIH diagnosis was not consistent with Friedman criteria. Patients with probable IIH or suggested IIH-WOP according to Friedman criteria were excluded as well as any patients with secondary causes of intracranial hypertension. We excluded pediatric IIH patients, defined as all patients diagnosed <14 years of age, as pediatric IIH is considered a distinct entity of the disease (11). The patient selection process is shown in Figure 1.

### 2.2 Data collection

Data were retrospectively extracted from electronically archived reports. We recorded the dates of clinical presentations, the date of the first diagnostic lumbar puncture (as a surrogate marker of the disease duration, defined as the time from the first diagnostic lumbar puncture to the date of the last visit). Baseline parameters were age, sex, weight, height, BMI and CSF-OP, as measured during lumbar puncture. All patients had CSF analysis, magnetic resonance imaging (MRI) of the brain and ophthalmological as well as neurological examination to exclude alternative causes of visual loss or intracranial hypertension. All patients were treated according to best practice including recommendation of weight loss for overweight or obese patients. We recorded the treatment strategy during the follow-up period, defining two groups of patients: (1) those who were treated pharmacologically (who received acetazolamide, topiramate and/or furosemide) and (2) those who were surgically managed by implantation of a ventriculoperitoneal (VP) shunt or by bariatric surgery. Symptoms and signs included the presence of (1) headache, (2) visual disturbance, (3) vertigo, (4) diplopia, (5) tinnitus, and (6) abducens palsy. Headache severity at baseline was recorded using the numeric rating scale (NRS, ranging from 0 to 10, where 0 is no pain and 10 refers to the most severe pain). Symptoms and signs, BMI, ophthalmologic findings and CSF-OP were recorded for follow-up visits.

### 2.3 Ophthalmologic findings

Best corrected visual acuity (BCVA) of the most severely affected eye was transformed to logMAR (logarithm of the minimum angle of resolution) for statistical analysis [logMAR = −log (decimal visual acuity)]. Visual field perimetric mean deviation (MD) in decibel [dB] was recorded with the Octopus 900 perimeter (Haag-Streit) using the 30–2° tendency-oriented perimetry. Visual fields required good reliability to be included (≤1/3 false negatives and ≤15% false positives). Visual field MD of less than −6.0 dB in at least one eye was considered abnormal. This threshold aligns with the Hodapp-Anderson-Parrish criteria for visual field grading and approximates the upper limit of inclusion used in the IIH Treatment Trial (−2 to −7 dB) (12, 13). Funduscopic papilledema grading of the most severely affected eye (i.e., the higher value) was extracted from ophthalmologic reports. Papilledema was graded according to the modified Frisén Scale from grade 0 (normal optic disc), 1 (minimal degree of edema), 2 (low degree of edema), 3 (moderate edema), 4 (marked edema) to grade 5 (severe papilledema) (14, 15). If Frisén grades were not in the reports, archived fundus photographs were reviewed. The presence of optic nerve atrophy was noted. All baseline ophthalmologic examinations were performed prior to lumbar puncture and prior to the initiation of therapy.

Long-term visual follow-up was recorded, if an ophthalmologic dataset including BCVA and the grade of papilledema, of at least 6 months after initial diagnosis was available. If more than one ophthalmologic dataset was available, the most recent dataset was included. Frisén grades were transformed into two categories for secondary analysis (16):

- mild and moderate papilledema (Frisén grades 1–3); and
- severe papilledema (Frisén grades 4 and 5).

Visual outcomes were assessed applying the following definitions:

- Persistent visual impairment: follow-up visual acuity ≥ 0.2 logMAR and/or perimetric MD of less than −6.0 dB in at least one eye.
- Ophthalmologic worsening (worsening of ophthalmologic findings): decline of visual acuity by ≥ 0.2 logMAR and/or occurrence of optic disc atrophy.

Worsening of papilledema in the absence of functional visual decline was not used to indicate poor visual outcome. As the presence of optic disc atrophy defined a poor visual outcome, patients with optic disc atrophy at baseline were excluded from the regression analysis.

### 2.4 Statistical analysis

Statistical analyses were performed using IBM SPSS Statistics software (IBM SPSS Statistics, Version 29.0. Armonk, NY: IBM Corp.). Categorical variables were expressed in frequencies and percentages, continuous normally distributed variables as mean ± standard deviation and non-parametric variables as median with interquartile range (IQR), as appropriate. Missing data was handled by listwise deletion and reporting of valid percentages. Q-Q plots and histograms were used to check for normal distribution of continuous variables. Univariate group comparisons were computed using Fisher’s exact test, two-sided t-test or Mann–Whitney U test, in non-parametric variables. Univariable correlation was analysed by Pearson test. Linked group comparisons of continuous variables were conducted with paired samples t-test or Wilcoxon-test, if not normally distributed. Univariable regression was applied to explore the association between baseline parameters and visual outcome. Backward stepwise binary logistic regression (likelihood-ratio) was conducted to identify possible predictors of a poor visual outcome (as defined above). The following candidate variables were included into the saturated backward regression model: baseline BMI, age at diagnosis, sex, disease duration, baseline CSF-OP, baseline severity of papilledema. At each step, variables that had the lowest contribution were removed from the model, i.e., with an elimination criterion set at a probability ≥ 0.1. Results of the regression analysis are presented as odds-ratio (OR) and 95% confidence intervals (95%CI). Goodness-of-fit was assessed using the Hosmer-Lemeshow-test. The level of significance was set at a two-sided *p*-value < 0.05. Prior to logistic regression, we assessed collinearity of the independent variables using Spearman’s rank correlation and calculation of variance inflation factors (VIF).

## 3 Results

### 3.1 Cohort characteristics

The IIH study cohort comprised 191 patients (Figure 1). Seven patients (4%, *n* = 7/191) were diagnosed with IIH-WOP and 184 with IIH (96%, *n* = 184/191). Cohort characteristics at baseline are presented in Table 1. Patients were predominantly female (86.4%, *n* = 165/191, males: 13.6%, *n* = 26/191). Mean age at diagnosis was 35.5 ± 12.5 years (range: 14–75). Men were significantly older than women at the time of diagnosis (*p* = 0.001).

**Table 1:** Baseline characteristics of the study cohort.

	All patients	Females	Males	*p*-value
	*n* = 191	*n* = 165	*n* = 26	
Age at diagnosis	35.5 ± 12.5	34.4 ± 11.6	42.8 ± 15.2	**0.001** ^**1**^
BMI [kg/m^2^]	34.4 ± 8.4	34.7 ± 8.3	32.4 ± 8.8	0.20^1^
CSF-OP [cmCSF]	36.2 ± 8.1	36.3 ± 8.4	34.8 ± 7.6	0.40^1^
Symptoms at baseline				
Headache	150/179 (84%)	137 (83%)	13 (50%)	**0.007** ^**2**^
Visual disturbance	155/174 (89%)	135/152 (89%)	20/22 (90%)	1.0^2^
Abducens palsy	18 (9%)	16 (9.7%)	2 (7%)	0.9^2^
Vertigo	32/71 (45%)	28/63 (44%)	4/8 (50%)	1.0^2^
Diplopia	31/80 (39%)	27/72 (38%)	4/8 (50%)	0.7^2^
Ophthalmologic findings				
BCVA [logMAR]	0.17 ± 0.38	0.17 ± 0.41	0.15 ± 0.15	0.90^1^
Frisén grade (median & IQR)	2 (1)	2 (1)	2 (1)	–
Optic disc atrophy	2	2	0	–

1 Two-sided *p*-values calculated by unpaired samples *t*-test.

2 Two-sided *p*-values obtained by Fisher’s exact test.

*P*-values in bold are statistically significant.

Baseline data on weight and height was available in 99% (*n* = 189/191) of patients. The mean BMI at baseline was 34.4 ± 8.4 kg/m^2^ (range: 20–80). Nineteen patients (10%) had a normal BMI (i.e., <25 kg/m^2^) at baseline. Forty patients were overweight (21%, *n* = 40/189, BMI: 25–30 kg/m^2^). Out of 69% (130/189) obese patients (BMI > 30 kg/m^2^), 42 patients had a BMI > 40 kg/m^2^. Mean CSF-OP at diagnosis was 36.2 ± 8.1 cmCSF. BMI and CSF-OP did not significantly differ between women and men. Baseline BMI and CSF-OP showed a moderate correlation (Pearson correlation, *r* = 0.33, *p* = 0.002). Visual disturbance (89%) and headache (84%) were the most common symptoms at baseline. Headache was more frequently reported by women (*p* = 0.007). Median baseline headache intensity on the NRS was 6 (IQR: 0–8). CSF-OP did not differ between patients with or without headaches (*p* = 0.35).

Diplopia was reported by 39%, vertigo by 45% of patients and 9% had abducent nerve palsy, each symptom or sign similarly frequent between women and men (Table 1). Headache and visual disturbance were less frequent at follow-up (headache: 68%, *p* = 0.02, visual disturbance: 55%, *p* = <0.001, Wilcoxon-test). Follow-up BMI was only available for 36 out of 90 patients, with the mean follow-up BMI being lower compared to baseline (*p* = 0.03). Mean CSF-OP at follow-up was lower than at baseline (*p* < 0.001, follow-up CSF-OP available in 42 of 90 patients).

### 3.2 Treatment

For the analysis of visual outcomes, follow-up data of 90 patients was available. During the follow-up period 17% were managed surgically by either implantation of a VP shunt (14%, 13/90) or by bariatric surgery (2%, 2/90). None of the patients were treated by sinus stenting. The group of surgically treated patients constituted of two men and 13 women. Surgical treatment was similarly frequent in men and women (men: 22%, *n* = 2/9, and women: 16%, 13/81; *p* = 0.71). Surgical treatment was more frequent in patients with moderate papilledema at baseline (22%) than in patients with mild (5%) and with severe papilledema (14%) at baseline. Data on medication were available in 83/90 (92%) patients. Of those, 62% were treated pharmacologically (i.e., received either/or a combination of: acetazolamide, topiramate and furosemide). Forty-five percent (54%) of the pharmacologically treated patients received acetazolamide in a median daily dose (MDD) of 1,000 mg, 23% received topiramate (MDD: 100 mg) and 8% furosemide (MDD: 40 mg).

### 3.3 Ophthalmologic findings and visual outcome

At baseline 184 of 191 patients had papilledema. In the whole study cohort, median Frisén grade was 2 (range: 0–5, IQR: 2) and median decimal BCVA at baseline was 0.8 (IQR: 0.4). Two patients had optic disc atrophy at baseline. Assessment of visual outcomes required the availability of ophthalmologic follow-up examinations at a minimum of 6 months after IIH diagnosis. Visual outcomes were available in 90 patients (i.e., the follow-up cohort), which represented 47% of the whole study cohort. Patients who were lost to follow up were older than patients in the follow-up cohort. Other baseline characteristics (sex, BMI, CSF-OP, BCVA, Frisén score) of the patients who were lost to follow-up did not significantly differ from those in the follow-up cohort (Supplementary Table 2).

The median time between the first and the last visit, i.e., the follow-up time was 2.0 years (range: 0.5 to 16, IQR: 4.2, mean: 3.7 ± 3.5). Overall visual acuity was significantly better at follow-up than at baseline (*p* = 0.03). Follow-up perimetric MD was documented in 52% (47/90) of patients and was lower than -6 dB in 40% (19/47). Perimetric MD was better at follow-up, compared to baseline data, although not statistically significant (*p* = 0.16). Median papilledema grades at follow-up were lower than at baseline (*p* < 0.001). One patient in the follow-up cohort had optic disc atrophy at baseline, eleven patients (12%) had optic disc atrophy at follow-up. Ophthalmologic characteristics of the follow-up cohort are presented in Table 2.

**Table 2:** Ophthalmologic findings at baseline and at follow-up in the follow-up cohort (90 patients).

	Baseline	Follow-up	*p*-value
BCVA [logMAR]^a^ (mean ± SD)	0.13 ± 0.20	0.077 ± 0.13	**0.03** ^**1**^
Frisén grade^b^ median (IQR)	2 (2)	0 (2)	**<0.001** ^**2**^
Visual field MD^c^ (mean ± SD)	7.3 ± 7.4	4.8 ± 4.9	0.16^1^
Optic disc atrophy *n*	1	11	–

SD, standard deviation; IQR, interquartile range.

1 Two-sided *p*-values calculated by paired samples *t*-test.

2 Two-tailed *p*-values obtained by Wilcoxon-test.

a Documented in 68 patients.

b Out of 79 patients.

c Documented in 47 patients.

*P*-values in bold are statistically significant.

Poor visual prognosis occurred in 30% (*n* = 27/90) of patients. This was defined as either (1) ophthalmologic worsening (decline of BCVA and/or occurrence of optic disc atrophy), which occurred in 12% and/or (2) persistent visual impairment (BCVA ≥ 0.2 logMAR and/or perimetric MD of less than −6.0 dB in at least one eye), which occurred in 27%.

In univariate group comparisons, neither sex, age, disease duration nor BMI and baseline CSF-OP were significantly different between the visual outcome groups (Supplementary Table 1). Neither the presence of headache at baseline (*p* = 1.0) nor the presence of abducens palsy (*p* = 0.4) were associated with the visual outcome. Pharmacologic treatment was similarly frequent in the group of patients with poor and without poor outcome (*p* = 1.0). However, surgical treatment was more frequently employed in patients with poor outcome (37% vs. 8%, *p* = 0.001, Supplementary Table 1).

In univariate regression analysis, surgical intervention was significantly associated with an increased risk of poor visual outcome (OR: 6.8, *p* = 0.002, Table 3). As surgical therapy is typically reserved for patients with more severe or rapidly worsening disease, this association reflects confounding by indication rather than a direct causal effect. In a multivariable model as a sensitivity analysis including both papilledema grade and surgical intervention, the associations for papilledema grading and surgical therapy remained significant. To avoid overadjustment and preserve the interpretability of the primary model, surgical intervention was not included in the main multivariable analysis.

**Table 3:** Regression analysis of the follow-up cohort (*n* = 90).

	Poor visual outcome			
	Univariable		Multivariable	
	OR (95% CI)^1^	*p*-value	OR (95% CI)^2^	*p*-value
Age at diagnosis	1.0 (0.97–1.05)	0.78	Not retained	–
Baseline BMI	0.98 (0.92–1.04)	0.53	Not retained	–
Baseline CSF-OP	1.03 (0.97–1.08)	0.37	Not retained	–
Disease duration	1.01 (0.99–1.02)	0.07	**1.02 (1.00–1.03)**	**0.03**
Male sex	3.35 (0.82–14)	0.09	**8.7 (1.7–44)**	**0.009**
Severe papilledema	4.44 (0.89–22)	0.07	**7.7 (1.4–43)**	**0.02**

CSF-OP, cerebrospinal fluid opening pressure; OR, odds ratio; 95%CI, 95% confidence interval.

1 Obtained by univariable binary regression.

2 Obtained by multivariable stepwise backward regression (likelihood ratio).

Bold values indicate significant likelihood of the independent variables contributing to a poor visual prognosis (Nagelkerke R-square: 0.25; Hosmer-Lemeshow test: *p* = 0.15).

Multivariable stepwise backward regression was utilized to explore possible predictors to the visual outcome: The saturated model included the following independent variables: sex, baseline BMI, age at diagnosis, baseline CSF-OP, disease duration in months, and severity of papilledema at baseline. Due to listwise deletion of missing data, multivariable analysis included 72 patients. The final model included papilledema grades at baseline, sex and disease duration (Table 3, Nagelkerke R-square: 0.25). Male sex (OR: 8.7, *p* = 0.009) and the presence of severe papilledema at baseline were associated with a greater likelihood for poor visual outcome (OR: 7.7, *p* = 0.02). Hosmer-Lemeshow-test indicated a good model fit (*p* = 0.15). Age and disease duration (*r* = −0.25), as well as ICP and papilledema grade (*r* = 0.16), showed weak pairwise correlations, and all variance inflation factor (VIF) values were close to 1.0, indicating no relevant multicollinearity among the independent variables in the regression model.

There was no significant difference in disease duration between male and female patients (median & IQR, males: 18 [45] months vs. females: 39 [55] months; *p* = 0.12).

## 4 Discussion

The aim of this study was to evaluate baseline characteristics, the visual prognosis and predictors of poor visual outcome in a well-characterized cohort of IIH patients in Germany.

In our study, the mean age at diagnosis was 35.5 years, which is slightly higher than reported in other observational cohorts (27–34 years) (4, 6, 17, 18). Our study excluded pediatric IIH patients, which is in line with prior studies (4). The overall demographic profile of our cohort closely resembled previous reports: IIH predominantly affected obese women, with 69% of our patients meeting criteria for obesity and only 7% being male. The frequency of typical signs and symptoms, as well as baseline BMI and CSF opening pressure, were likewise comparable to other published cohorts (17, 19, 20).

During follow-up, patients demonstrated significant improvement in papilledema and visual acuity, yet 10 patients developed optic disc atrophy. A poor visual outcome was observed in 30% of the cohort, which is consistent with the wide range (10–60%) described in earlier studies, though definitions of visual deterioration vary considerably across reports (2, 6, 17, 21, 22). For instance, Wall et al. (12) reported that 32% of IIH patients had permanent visual field loss, while Corbett et al. (23) observed severe visual impairment in nearly 10% of cases (23, 24). The proportion of patients receiving surgical intervention in our study (17%) was also within the range of previous cohorts (2–20%) (17, 20, 25). Bruce et al. found that 50% of men with IIH developed moderate to severe permanent vision loss compared to only 30% of women (26). Possible explanations include delayed diagnosis in men, less frequent headache symptoms, and atypical presentation, which may contribute to more advanced optic nerve damage at presentation.

Our multivariable regression identified male sex and severe papilledema at baseline as significant predictors of poor visual outcome. In a multicenter study of 721 IIH patients, men were more likely than women to develop severe visual loss, with a risk ratio of about 2.1 for at least one eye (26). This finding has been replicated, with delayed diagnosis and atypical presentation (e.g., fewer headaches) cited as contributing factors (4, 26, 27).

Papilledema severity emerged as a predictor of outcome in our study, consistent with prior reports: In the Idiopathic Intracranial Hypertension Treatment Trial, patients with high-grade papilledema had an odds ratio of 8.66 for significant visual field loss compared to those with lower grades (28). Patients presenting with Frisén grade 4–5 or atrophy were more likely to have poor visual outcomes (29). Interocular comparisons in patients with asymmetric papilledema showed that the eye with higher-grade papilledema consistently has worse visual function across multiple modalities, including visual field mean deviation and central acuity, with peripheral field loss being most pronounced (30). In contrast, and in line with other reports, neither BMI nor CSF opening pressure were associated with visual outcomes in our cohort (4).

Taken together, our real-world data confirm the observations made in prior clinical trials and further highlight the importance of timely recognition and risk stratification in IIH. Importantly, while severe papilledema is a marker of increased risk, at this stage vision may already be compromised, which emphasizes the need for earlier predictors of visual decline.

There are limitations given the retrospective nature of our study. All data was collected during routine clinical work-up and some patients were lost to follow-up. Hence, asymptomatic patients or patients with a mild disease course were more likely to discontinue management than to be expected in a prospectively collected cohort. The proportion of more severely affected patients may therefore be overestimated. Moreover, the use of a fixed MD threshold to define poor visual outcome may have been overly stringent and could have excluded patients with milder but relevant impairment. Additionally, repeat visual field testing (which was used in the IIH Treatment Trial) to confirm worsening of visual fields was not performed (12). We did not account for the learning effect in perimetry, and therefore some improvement in visual fields over time may be attributable to this effect. However, only visual fields with good reliability were included in the analysis.

A further limitation is that individual follow-up periods were variable. We diligently applied the current diagnostic criteria and minimized the risk of confounders, choosing a backward regression model and taking treatment strategies and disease duration into account. As this is a real-world cohort, treatment regimens may have varied regarding intensity and the time of initiation, potentially introducing bias. Adjusting regression models for more detailed treatment parameters (i.e., specific drugs and dosages, time between the diagnosis and initiation of therapy) would have caused overfitting. Lastly, patient reported outcomes, such as headaches, were not well assessed in this cohort. Similarly, the discrepancy between the reported rate of diplopia and the observed frequency of abducens nerve palsy reflects the difference between symptom-based reporting and examination-based findings in routine clinical records, limiting the completeness of our risk factor assessment.

## 5 Conclusion

In this first retrospective, real-world cohort of German IIH patients, overall visual outcomes were generally favorable, though a subset developed persistent visual impairment or deterioration in visual acuity. Consistent with international studies, male sex and severe papilledema at diagnosis emerged as significant risk factors for poor visual prognosis, while age, BMI, and CSF opening pressure showed no association. These findings provide important region-specific data, emphasize the need for early recognition and risk stratification, and highlight the importance of identifying predictors of outcome before irreversible optic nerve damage occurs.

## Data Availability

The raw data supporting the conclusions of this article will be made available by the authors, without undue reservation.
