# Supplementary material for: Visual prognosis in idiopathic intracranial hypertension: observations from a retrospective cohort in Germany
Source: Front Neurol. 2025 Nov 12;16:1698486. doi: 10.3389/fneur.2025.1698486 (PMC12647045; doi:10.3389/fneur.2025.1698486)
Supplement: Supplementary file 1 [file Table_1.docx]

**Supplemental Table 1.** Group comparisons of clinical characteristics and visual outcomes.

|  |  | | |  | poor visual outcome | | | | | |
| --- | --- | --- | --- | --- | --- | --- | --- | --- | --- | --- |
|  | **poor visual outcome** | | |  | **ophthalmologic worsening^3^** | | | **persistent visual impairment** | | |
|  | **yes**  n = 27 | **no**  n = 63 | ***p*-value** |  | **yes**  n = 11 | **no**  n = 78 | ***p*-value** | **yes**  n = 24 | **no**  n = 66 | ***p*-value** |
| **age at diagnosis**  years (mean ± SD) | 33.8 ± 12.5 | 33.1 ± 10.4 | 0.80^1^ |  | 31.7 ± 15.5 | 33.7 ± 10.3 | 0.69^1^ | 34.3 ± 12.9 | 33.0 ± 10.3 | 0.67^1^ |
| **baseline BMI**  kg/m^2^ (mean ± SD) | 33.8 ± 8.9 | 34.9 ± 7.4 | 0.58^1^ |  | 33.4 ± 9.6 | 34.7 ± 7.7 | 0.66^1^ | 33.5 ± 8.8 | 34.9 ± 7.5 | 0.50^1^ |
| **baseline CSF-OP**  cmCSF (mean ± SD) | 37.1 ± 8.8 | 35.4 ± 8.1 | 0.39^1^ |  | 39.1 ± 8.0 | 35.3 ± 8.2 | 0.17^1^ | 36.3 ± 8.7 | 35.8 ± 8.2 | 0.83^1^ |
| **disease duration**  months (mean ± SD) | 69.6 ± 62.9 | 48.2 ± 41.2 | 0.11^1^ |  | 59.7 ± 46.5 | 54.1 ± 50.2 | 0.72^1^ | 72.4 ± 65.3 | 48.1 ± 40.8 | 0.10^1^ |
| **therapeutic strategy**   - **surgical** - **pharmacological^4^** | 37% (10/27)  58% (14/24) | 8% (5/63)  61% (36/59) | **0.001^2^**  1.0^2^ |  | 27% (3/11)  50% (5/10) | 14% (11/78)  63% (45/72) | 0.37^2^  0.5**^2^** | 42% (10/24)  67% (14/21) | 8% (5/66)  58% (36/62) | **<0.001^2^**  0.61**^2^** |
| **sex**   - **male** - **female** | 19% (5/27)  81% (22/27) | 6% (4/63)  94% (59/63) | 0.09^2^ |  | 9% (1/11)  91% (10/11) | 10% (8/78)  90% (70/87) | 1.0^2^ | 21% (5/24)  79% (19/24) | 6% (4/66)  94% (62/66) | 0.053^2^ |
| CSF-OP: cerebrospinal fluid opening pressure. SD: standard deviation.  ^1^two-sided p-values calculated by independent samples t-test.  ^2^calculated by Fisher’s exact test.  ^3^only available for 89 patients. The missing values in the “ophthalmologic worsening” group is due to lack of baseline visual acuity in 1 patient.  ^4^data on medical therapy was available for 83 out of 90 patients in the outcome analyses. | | | | | | | | | | |

**Supplemental Table 2.** Baseline characteristics of the patients lost to follow-up and patients in the follow-up cohort.

|  | follow-up cohort  (n = 90) | patients lost to follow-up  (n = 101) | p-value |
| --- | --- | --- | --- |
| **age** | 33.3 ± 11.0 | 37.4 ± 13.4 | 0.02^1^ |
| **female/male** | f = 81; m = 9 | f = 84; m = 17 | 0.21^2^ |
| **BMI [kg/m^2^]** | 34.6 ± 7.8 | 34.3 ± 9.0 | 0.81^1^ |
| **CSF-OP [cmCSF]** | 35.9 ± 8.3 | 36.3 ± 7.9 | 0.73^1^ |
| **Frisén grade**  **(median & IQR)** | 2 (2) | 2 (1)^3^ | 0.21^4^ |
| **BCVA [logMAR]** | 0.13 ± 0.20 | 0.17 ± 0.37 | 0.36^1^ |

^1^two-sided p-values calculated by independent samples t-test.

^2^calculated by Fisher’s exact test.

^3^available in 73 patients.

^4^calculated using Mann-Whitney-U-test.
